# Does the world need a scientific society for research on how to improve healthcare?

**DOI:** 10.1186/1748-5908-7-10

**Published:** 2012-02-29

**Authors:** Michel Wensing, Jeremy M Grimshaw, Martin P Eccles

**Affiliations:** 1 Scientific Institute for Quality of Healthcare, Radboud University Nijmegen Medical Centre, Nijmegen, Netherlands; 2 Clinical Epidemiology Program, Ottawa Health Research Institute, Ottawa, Canada; 3 Institute of Health and Society, Newcastle University, Newcastle upon Tyne, UK

## Abstract

In this editorial, we reflect on the arguments for starting a scientific society focused on research on how to improve healthcare. This society would take an inclusive approach to what constitutes healthcare. For instance, it should include mental health healthcare, treatment for substance abuse, the work of allied health professions, and preventive healthcare. The society would be open to researchers from all traditions. Thus, we take an inclusive approach to what constitutes scientific research, as long as it uses rigorous methods, is focused on improving healthcare, and aims at knowledge that can be transferred across settings. The society would primarily target scientific researchers but would invite others with an interest in this area of research, regardless of their discipline, position, field of application, or group affiliation (*e.g.*, improvement science, behavioral medicine, knowledge translation). A society would need fruitful collaboration with related societies and organizations, which may include having combined meetings. Special links may be developed with one or more journals. A website to provide information on relevant resources, events, and training opportunities is another key activity. It would also provide a voice for the field at funding agencies, political arenas, and similar institutions. An organizational structure and financial resources are required to develop and run these activities. Our aim is to start an international debate, to discover if we can establish a shared vision across academics and stakeholders engaged with creating scientific knowledge on how to improve healthcare. We invite readers to express their views in the online questionnaire accessed by following the URL link provided at the end of the editorial.

## Background

In the previous decades, scientific research on “how to improve healthcare” has been increasingly recognized as a legitimate field of research [1,2]. It has evolved under various names, including implementation science, knowledge translation (KT) research, improvement science, evidence-based practice, research utilization, delivery science, and patient safety science [3]. Also across a range of other academic fields, such as clinical epidemiology, medical education, and clinical sciences, researchers have started to pay attention to questions concerning how to improve healthcare. Dedicated scientific journals have emerged, such as *Implementation ScienceBMJ Quality and Safety*, and the *International Journal for Quality in Health Care*. These developments have occurred across the world, although not at equal speed and shape across countries, facilitated by major health-research funders, such as the Canadian Institutes of Health Research, ZonMW in The Netherlands, the Agency for Healthcare Research and Quality, and (more recently) the National Institutes of Health in the United States [4]. Policy makers at the highest level are calling for more and better research in the area [5-7].

From our perspective, as academics engaged with improving healthcare, these developments are very positive. Whilst we continue to have debates on the nomenclature, epistemology, concepts, methods, and ways forward for the field, we share the same ambition. Our core idea is that we need to use a scientific process to understand how knowledge is translated into healthcare practice, management, and policy to achieve the best possible (health) outcomes at the optimum value. This implies that we want to learn about the needs of research users and address those needs. Implementation science has been defined as “the study of the methods and results of the implementation of proven treatments, practices, organizational and management interventions into routine practice” [8]. The variety of other names attached to the field bring their own nuances to the area [9], but the reality is that there are far more commonalities in the research conducted under these different names than differences. For instance, the focus on “proven” interventions in implementation science is relative because the applicability of research evidence in a specific setting may be at stake, new research can change what is regarded proven, and interventions may be adapted when implemented, which may influence their “proven” effectiveness.

We firmly believe that our research serves the needs of decision makers in healthcare—health professionals, managers, policy makers, and, indeed, also patients—aiming at improving outcomes of healthcare for patients and societies. Our research contributes to the sustainability and productivity of healthcare systems, which is increasingly important given current global challenges of scarce resources and increasing demands. Many of us focus on specific health-profession groups, healthcare organizations, or defined clinical area (as clinicians, managers, or policy makers). This is crucial for having impact on clinical, organizational, and policy decisions and practices, but it has also led to a fragmentation across various domains of healthcare and health sciences, as well as in fields beyond healthcare. We believe that this situation reduces progress in this academic field. It is a strength that we have heterogeneous backgrounds and research training, but it also adds to the fragmentation and the lack of a comprehensive and integrated strategic research agenda to advance scholarship in the area. For instance, we attend a wide range of conferences and training programs, and many of these lend themselves to practical application rather than scientific research. Some countries have established dedicated platforms for our field (*e.g.*, United States and Canada), but in other countries such platforms are lacking.

Thus, we believe that creating international links between researchers to enhance scientific discussion would enhance the field. Setting up an international society focusing on the conduct of science in this area is one way to facilitate these linkages. In this editorial, we now reflect on the arguments for starting such a scientific society. Our aim is to start an international debate, to discover if we can establish a shared vision across academics and stakeholders engaged with providing scientific knowledge on how to improve healthcare.

## What could a scientific society achieve?

The objectives and activities of an international scientific society could include the following:

1. To provide an international forum for scientific exchange, which may take the form of traditional conferences, focused working groups, and online discussion networks. This would contribute to the quality of the science and also help individuals to develop an identity as a researcher in this field. It may also be possible to establish explicit links to dedicated scientific journals in the field.

2. To announce relevant existing training and development opportunities in the field and possibly also facilitate such training programs, particularly for young researchers. A journal, website, or online mailing group would be needed for this purpose. Providing information on relevant conferences, funding opportunities, and job vacancies could be further services provided by a scientific society.

3. Help researchers (in any field) to disseminate their research findings more effectively to stakeholders and raise awareness about determinants of improvements in healthcare practice, management, and policy.

4. Promote and lobby for our science across the world, particularly in countries where it is currently underdeveloped, so that it reaches a critical mass, which is needed to survive and flourish. In particular, a society could help develop core competencies for scientists [10], lobby for career paths in academic and healthcare structures for scientists and practitioners, and lobby for sustained research funding to support implementation science.

## Initial considerations

Drafts of this editorial were sent to all editorial board members and editors of the journal *Implementation Science* and a few others. A total of 31 individuals (including 28 of 56 members of the *Implementation Science* editorial board and editors; see Acknowledgment) provided almost 8,700 words of comment. While many had a positive feeling about the idea of setting up a scientific society, a few were concerned that a new scientific society might create a new silo of vested interests that blocks rather than promotes progress. One individual suggested that a separate society might effectively reduce attention for the field in other organizations and meetings. Some individuals pointed to other organizations and networks that they felt overlapped with our idea for a potential new scientific society. However, no one identified an existing international organization with a primary focus on research on how to improve healthcare. Thus, there would be the issue of how to position a scientific society in relation to existing national and international organizations, networks, and meetings in the field that in part share a common interest, such as (but not limited to) those for quality improvement, evidence-based practice, education of health professionals, healthcare management, patient safety, health services research, health policy, and health systems. There was a feeling that developing linkages with those organizations would be important.

Amongst the individuals with positive feelings about setting up an international society, a range of issues of scope were discussed. Many commentators supported a focus on research, given the more applied focus of most existing organizations and meetings (including those focused on quality improvement, knowledge transfer, and knowledge implementation). One issue was how to include decision makers (clinicians, managers, and policy makers) in a scientific society, an issue that was regarded as very important by some contributors. Some comments emphasized the importance of practice and partners in implementation and improvement, suggesting that research should be de-emphasized. Several individuals emphasized the importance of including healthcare management and health policy, in addition to healthcare practice, as key domains for improving healthcare. A few commentators argued for extension of scope beyond healthcare to include, for instance, social care and the organizational sciences. Some felt strongly engaged with improvement science, others with implementation science, but the difference between the two was not clear.

There were mixed feelings about the question of whether a separate meeting was needed for such a society, in addition to the many meetings that already exist. One suggested option was to link to or integrate with existing meetings rather than setting up a new meeting.

## Outline of a society

Among the responders to our proposals, there seemed to be a broad interest in exploring the idea of setting up an international scientific society for research on how to improve healthcare. However, a number of issues seemed contested or controversial. In this section, we sketch an outline of a possible international society. We emphasize that this proposal reflects the authors’ thinking and not necessarily the ideas of all who contributed ideas to an earlier version of the editorial. Its purpose is to generate a wider debate.

The proposed society would focus on research on how to improve healthcare. It would take an inclusive approach to what constitutes healthcare. For instance, it should include mental health healthcare, treatment for substance abuse, the work of allied health professions, and preventive healthcare. The society would be open to researchers from all traditions, including positivistic, realist, and constructivist approaches. Thus, we take an inclusive approach to what constitutes scientific research, as long as it uses rigorous methods, is focused on improving healthcare, and aims at knowledge that is potentially transferrable across settings and contexts. The society would primarily target scientific researchers but would invite others with an interest in this area of research, regardless of their discipline, position, field of application, or group affiliation (improvement science, behavioral medicine, knowledge transfer, etc.).

The society would have individual researchers as members and potentially also organizations. A further possibility is to create special memberships, for instance, for trainees and for experienced researchers (such as fellowships). All members would pay a membership fee. Having meetings for scientific exchange every one or two years would be a key activity of the society. Such meetings could be independent or linked to existing international conferences and organizations. A society would need fruitful collaboration with related societies and organizations, which may include having combined meetings. A website to provide information on relevant resources, events, and training opportunities is another key activity. An organizational structure and financial resources are required to develop and run these activities.

## Next steps

The proposed society will only come into existence if it gets the international support from researchers, funders, and stakeholders. We invite readers (of any background) to express their views in the online questionnaire accessed by following the URL link provided below (this will remain open until July 2012). It is unlikely that we will achieve consensus on all items, but we hope to find common ground that enables us to take further steps, including the organization of an inaugural meeting. We believe that we need a society to have a space for scientific debate, but this will only be viable if this belief is widely shared.

**URL link:**https://www.iq-surveys.nl/index.php?sid=93665&lang=en

## Authors’ contributions

MW led the writing, and MPE and JMG provided detailed comments and redrafts. The contributions of the listed contributors were incorporated into the text by MW. All authors read and approved the final manuscript

## Competing interest

MPE is Editor in Chief of *Implementation Science*, MW is an Associate Editor, and JMG is a member of the Editorial Board.

## References

[B1] Grol R Berwick DM Wensing M On the trail of quality and safety in health care BMJ 2008 336 74 76 10.1136/bmj.39413.486944.AD 18187724 PMC2190232

[B2] Remme JHF Adam T Becerra-Posada F D’Arcangues C Devlin M Gardner C Ghaffar A Hombach J Kengeya KFK Mbewu A Mbizvo MT Mirza Z Pang T Ridley RG Zicker F Terry RF Defining research to improve health systems PLoS Med 2010 7 e1001000 10.1371/journal.pmed.1001000 21124816 PMC2993153

[B3] Straus SE Tetroe J Graham I Defining knowledge translation CMAJ 2009 181 165 168 10.1503/cmaj.081229 19620273 PMC2717660

[B4] Tetroe JM Graham ID Foy R Robinson N Eccles M Ward J Wensing M Durieux P Légaré F Nielson CP Adily A Porter C Shea B Grimshaw J Health research funding agencies’ support and promotion of knowledge translation: an international study Milbank Q 2008 86 125 155 10.1111/j.1468-0009.2007.00515.x 18307479 PMC2690338

[B5] Six-third World Health Assembly **WHO’s role and responsibilities in health research**. Document A63/21 approved on May 21, 2010 http://apps.who.int/gb/ebwha/pdf_files/WHA63/A63_R21-en.pdf

[B6] Pan American Health Organization **PAHO’s Policy on Research for Health**. Approved at the 61st session of the Directing Council, 2009 http://new.paho.org/hq/dmdocuments/2010/RESEARCHpolicyBKLETeng_web.pdf

[B7] European Science Foundation **Implementation of medical research in clinical practice.** ESF, 2011 http://www.esf.org/index.php?eID=tx_nawsecuredl&u=0&file=fileadmin/be_user/research_areas/emrc/FL/FLIP/8._Implem_MedReseach_ClinPractice_FINAL.pdf&t=1325850816&hash=53a69b34a78ab42904f563f571e77ec8c6049c37

[B8] Eccles MP Mittman BS Welcome to Implementation Science Implem Sci 2006 1 1 10.1186/1748-5908-1-1

[B9] McKibbon KA Lokker C Wilczynski NL Ciliska D Dobbins M Davis DA A cross-sectional study of the number and frequency of terms used to refer to knowledge translation in a body of health literature in 2006: a Tower of Babel? Implem Sci 2010 5 16 10.1186/1748-5908-5-16 PMC2834600 21080976

[B10] Straus SE Brouwers M Johnson D Lavis JN Legare F Majumdar SR McKibbon KA Sales AE Stacey D Klein G Grimshaw J Stihr KC Core competencies in the science and practice of knowledge translation: description of a Canadian strategic training initiative Implement Sci 2011 6 127 10.1186/1748-5908-6-127 22152223 PMC3292943

